# Novel Silicone Rubber–Based Multi-Dimensional Filler Composite Electrode Materials for the Dielectric Elastomer Actuation Technology of Micro-Crawling Robots

**DOI:** 10.3390/polym18131561

**Published:** 2026-06-23

**Authors:** Yang Hong, Yun Yang, Zening Lin, Tao Jiang, Zirong Luo

**Affiliations:** 1 College of Intelligence Science and Technology, National University of Defense Technology, Changsha 410073, China; hongyang16@nudt.edu.cn (Y.H.);; 2 National Key Laboratory of Equipment State Sensing and Smart Support, National University of Defense Technology, Changsha 410073, China

**Keywords:** soft electrode materials, composite electrode materials, multi-dimensional conductive fillers, dielectric elastomer actuators, micro-crawling robots

## Abstract

Aiming to develop high-performance flexible electrode materials for dielectric elastomer actuation systems applied to micro-crawling robots, this study proposes multi-dimensional filler composite electrode materials with a methyl vinyl silicone rubber matrix. Three types of conductive fillers—namely, zero-dimensional super-conductive carbon black, one-dimensional single-walled carbon nanotubes, and two-dimensional flaky micron-sized silver powder—were employed to construct a hierarchical multi-dimensional conductive network within the silicone rubber matrix via a three-stage fabrication strategy. The electrical conductivity and conductive stability of the as-prepared composite electrode materials were systematically investigated, where the intrinsic mechanisms and evolutionary laws of material electrical performance variations were analyzed. Furthermore, the effects of fillers with different dimensional morphologies on the comprehensive properties of the composites at each fabrication stage were explored, and the optimal filler dosage for each component was determined. Microstructural observations of the staged conductive network formation further verified the rationality of the stage-based functional design model. The optimized composite electrode delivers an initial electrical conductivity of 1.5 × 10^4^ S/m, with only a 14.9% conductivity attenuation under 50% tensile strain, demonstrating excellent electromechanical stability. Moreover, a prototype micro-crawling robot was fabricated using the optimized composite electrode, achieving a maximum linear crawling speed of 8 mm/s. These experimental results validate the feasibility and superiority of the proposed multi-dimensional filler composite strategy. This work provides a novel technical approach for the design and development of high-performance flexible electrode materials for flexible electronic and micro-robotic actuation applications.

## 1. Introduction

From the Paleolithic Age to the “Electrical Age” of the Second Industrial Revolution, humans have primarily focused on the research and application of hard materials. However, organisms that have evolved in nature over tens of thousands of years typically grow based on soft materials (such as muscles in organisms, stems and leaves in plants, etc.). Traditional hard materials include metals, rocks, ceramics, etc., while polymers are typical soft materials [1]. Some soft materials can respond to external stimuli and undergo effective deformations, such as stretching and bending, and are thus known as smart soft materials. These smart soft materials with special response capabilities find widespread applications in fields such as flexible electronics, biomedicine, soft robotics, aerospace, and other fields [2]. Smart soft materials exhibit abilities similar to those of real biological muscles when responding to external stimulus; they are also known as active soft materials or smart artificial-muscle materials [3,4,5].

As a smart soft active polymer material, dielectric elastomers (DEs) can undergo significant deformation under the influence of an external electric field. They possess advantages such as high strain energy density, high electromechanical conversion efficiency, rapid response, and being lightweight. These properties have led to their widespread application in areas like actuators, sensors, and energy harvesters. Furthermore, they exhibit immense potential for applications in fields like artificial muscles, soft robots, aerospace, and other fields [6,7,8,9,10,11,12,13].

**Figure 1:**
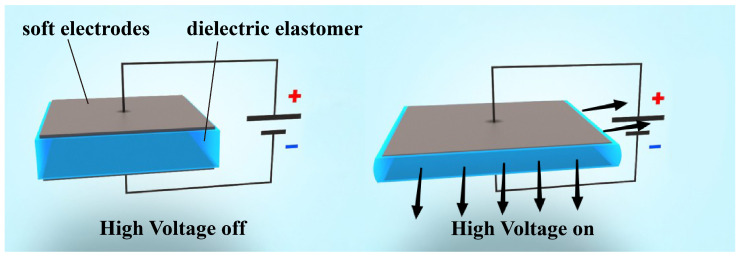
Schematic diagram of the electro-actuation deformation principle of dielectric elastomers.

As shown in Figure 1, the basic functional structure of a dielectric elastomer actuator (DEA) is sandwich-like, with the blue part in the middle being the dielectric elastomer layer, and the gray parts above and below being the flexible electrode layers, which need to possess excellent electrical properties and good mechanical properties [14]. When an external high-voltage electric field is applied to the electrodes on both sides, opposite charges on the electrodes attract each other while like charges on the electrodes on the same side repel each other, exhibiting an overall electrostatic attraction between the electrodes. This internal force squeezes the dielectric elastomer layer in the middle, forcing it to reduce in thickness and expand in area, which is the basic principle of DEAs [15].

The flexible electrode is one of the most critical components for achieving DEA functionality and flexibility, and it should possess excellent electrical properties, such as high conductivity and high conductivity stability, as well as mechanical properties, such as high compliance, high adhesion, and high stretchability [16]. A flexible electrode with high conductivity directly boosts overall DEA functionality in three key aspects [17]: 1. Enhanced Actuation Efficiency and Speed: High conductivity reduces electrode resistance, minimizing RC delay and Joule energy loss, enabling faster charge/discharge and higher-frequency actuation. For instance, Ag–EGaIn biphasic electrodes with high conductivity enable 5× faster actuation than carbon grease electrodes. 2. Uniform Electric Field and Maximum Strain: High conductivity ensures homogeneous charge distribution across the dielectric film, avoiding field concentration and enabling the DEA to achieve its theoretical maximum actuation strain. Low-conductivity electrodes often suffer from “dead zones” with insufficient charge, reducing effective deformation. 3. Improved Energy Efficiency and Durability: Lower resistance reduces heat generation during operation, mitigating thermal aging and breakdown risks, thus extending DEA service life. High conductivity also supports low-voltage operation, expanding application feasibility in portable and wearable devices [18]. Conductivity stability is particularly critical for DEA operation because DEAs undergo repeated large-strain deformation (often >100% area strain) under high electric fields (typically 10–100 V/μm) during long-term cycling [19]. Unstable conductivity (e.g., significant resistance increases under strain or over cycles) leads to non-uniform electric field distribution, localized Joule heating, and premature dielectric breakdown, severely limiting DEA lifespan and reliability [20]. For example, conductive fillers may detach or reorient under cyclic stretching, disrupting the conductive network and causing irreversible performance degradation. In contrast, electrodes with high conductivity stability maintain consistent electrical properties under deformation and cycling, ensuring stable actuation strain, force output, and long-term durability [21].

However, currently used flexible electrode materials often fail to combine excellent electrical and mechanical properties, which poses a major technical challenge for high-performance and long-life DEA devices [22]. The inherent performance trade-off severely restricts the practical engineering application of DEAs. Typical high-conductivity electrode materials—including thin metal films, pristine silver nanowire networks, and concentrated carbon nanotube films—can deliver excellent electrical conductivity and low interfacial resistance, but generally suffer from poor mechanical flexibility, low stretchability, weak interfacial adhesion, and high modulus rigidity. When such mechanically inferior electrodes are assembled into DEAs, they cannot synchronously and uniformly deform with the soft dielectric elastomer substrate under cyclic large-strain actuation. During repeated stretching and releasing cycles, rigid or low-toughness conductive layers are prone to microcrack initiation, structural fracture, conductive network disconnection, and interfacial delamination. These defects further cause progressive resistance surge, uneven surface-charge distribution, and localized electric field concentration on the DEA film. Severe electric field concentration will induce local thermal hotspots and premature dielectric breakdown, resulting in attenuated actuation strain, unstable output force, and a shortened service life of DEA devices. In extreme cases, mechanical fracture of the electrode will directly cause device failure and interrupt continuous actuation operation. In contrast, conventional highly compliant electrodes, such as carbon grease, possess favorable mechanical stretchability but are limited by low conductivity and poor cycling stability, failing to meet the requirements of high-efficiency and high-frequency DEA actuation.

Currently, flexible electrodes are mainly classified into three categories—metal-based, carbon-based, and composite flexible electrodes—each with distinct characteristics [23]. Metal-based flexible electrodes exhibit excellent electrical conductivity, yet they suffer from high bulk density, which hinders weight reduction [24]. Additionally, such electrodes entail relatively high costs. Their interior is prone to oxidation, and in severe cases, may lead to electrochemical corrosion [25]. More importantly, metal-based flexible electrodes possess a high Young’s modulus and poor resilience and tend to undergo irreversible deformation. These drawbacks make them inapplicable for DEAs [26]. Carbon-based flexible electrodes rely on carbon conductive materials, such as carbon black, graphene, carbon nanotubes, and carbon fibers, to achieve electrical conduction. They feature low density, superior chemical stability, and diverse structures, and can form stable dispersions with various organic solvents and active substances [27]. Nevertheless, the incorporation of nanoscale carbon fillers remarkably increases the hardness and modulus of composites and reduces flexibility [28]. Accordingly, the loading of carbon fillers is restricted, resulting in inferior electrical conductivity compared with metallic fillers. Furthermore, nanoscale carbon fillers have a high surface energy and tend to agglomerate in the matrix, leading to poor dispersion [22]. In summary, the above-mentioned flexible electrode materials generally fail to combine outstanding electrical and mechanical properties.

New-generation flexible electronic devices impose stringent requirements on electrode materials, including high flexibility, low modulus, low density, and superior conductive stability. Specifically, the matrix of flexible electrodes is expected to possess low hardness and a low Young’s modulus, as well as excellent resilience. Polymeric elastomers represented by rubbers exhibit prominent performance advantages. Through interactions between the vulcanization system and unsaturated bonds on the main and side chains of macromolecules, a densely cross-linked network with ample spatial structure and high elastic potential energy storage can be constructed, which serves as a fundamental guarantee for the elasticity of electrodes [29]. In terms of conductive systems, research no longer relies on networks composed of single large-sized conductive fillers. Instead, greater emphasis is placed on the hybrid incorporation of multi-dimensional fillers in diverse forms, and the synergistic effects generated between dual or multiple conductive networks have attracted extensive attention [30]. The main chain of silicone rubber consists of Si–O bonds. Its unique bond length and bond angle endow the material with exceptional flexibility. Among silicone rubber derivatives, polydimethylsiloxane (PDMS) is renowned as the “king of flexibility”. Accordingly, adopting silicone rubber as a typical flexible matrix enables the material to maintain a relatively low strength and modulus [31]. Additionally, silicone rubber features a wide service temperature range (−100 to 300 °C), oxidation resistance derived from its saturated main chain, stable processability, and excellent weather resistance. It is also non-toxic, odorless, and highly biocompatible. Hence, conductive materials based on silicone rubber can meet the application requirements of flexible wearable electronics, flexible energy storage devices, and biomedical sensors. A variety of related research achievements and products have been developed, demonstrating great application potential and promising development prospects. Pure silicone rubber without any conductive fillers possesses outstanding insulating properties. Incorporating fillers such as fumed silica, titanium dioxide, and titanate can endow silicone rubber with desirable dielectric properties, forming dielectric elastomers (DEs). The capacitance of DE materials can be regulated by adjusting parameters, including thickness, area, and dielectric constant. Liquid silicone rubber has a low molecular weight and viscosity. After curing, the resultant material features low strength and hardness with superior softness. It can be readily fabricated into thin films via a wide range of processing and molding methods [32,33,34], which perfectly conform to the flexibility requirements of the designed electrode materials. Therefore, in this article, novel silicone rubber–based multi-dimensional filler composite electrode materials were designed and prepared using methyl vinyl silicone rubber (VMQ) as the matrix. They possess excellent electrical and mechanical properties, fully meeting the performance requirements of DEAs for flexible electrode materials.

Conductive materials generally refer to a class of functional materials with conductivity above 10^0^ S/cm, capable of transmitting carriers in an electric field to conduct electricity [35]. Common conductive materials in the field of flexible electronics are primarily classified into metallic conductive materials, carbon-based conductive materials, and polymer conductive materials based on their chemical compositions [21,36,37]. Polymer conductive materials stand out as the most flexible, stretchable, and designable conductive composite materials among common flexible electrode materials. Therefore, developing novel polymer-based conductive composites based on the theory of polymer conductive materials is an effective approach to designing and preparing new flexible electrode materials that exhibit excellent flexibility, stretchability, and conductivity.

Theoretically, there exists a regular correlation between the bulk resistivity of polymer-based conductive composites and the loading content of a single type of conductive filler [38,39,40]. However, the conductive network can be affected by numerous factors during filler incorporation, including the size, morphology, and distribution of conductive filler particles within the resin matrix, as well as the matrix type, crystallinity, fabrication process, and curing conditions of the composites [18,41,42]. After the conductive path within the system is formed, carriers migrate along the conductive path or some of it to form conductivity. The most widely studied and applied types of conductive polymer materials with filler composites are carbon-based and metal-filled ones. However, incorporating a certain amount of carbon or metal fillers into polymer materials can affect their mechanical and processing properties [43]. To date, many researchers have attempted to develop composite conductive polymer materials with various fillers, but most of them suffer from the problem of uneven material properties. Therefore, finding a balance point that can strike a harmony among the conductivity, mechanical properties, and processing performance of composite material systems is an important research topic. This study proposes multi-dimensional filler composite electrode materials based on a silicone rubber matrix, and gradually endows them with excellent and relatively balanced material properties.

## 2. Materials and Methods

### 2.1. Methods

**Figure 2:**
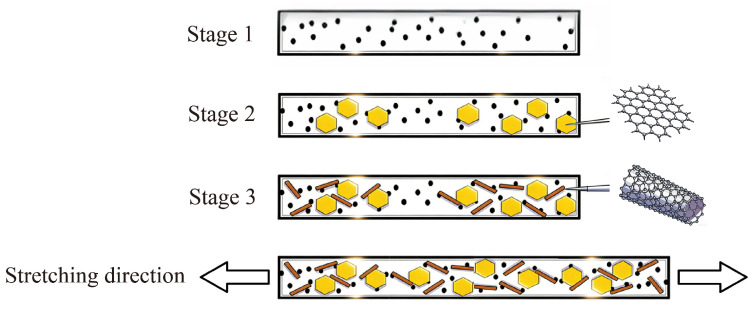
The staged functional design scheme for silicone rubber–based multi-dimensional filler composite electrode materials.

The silicone rubber–based multi-dimensional filler composite electrode materials utilize 110-2-type methyl vinyl silicone rubber (VMQ) as the matrix, and incorporate superconducting carbon black (SCCB), flaky micron-sized silver powder (FMSP), and single-wall carbon nanotubes (SWCNTs) as conductive fillers. The filling process with different types of conductive fillers is completed in three stages to optimize the performance of the electrode material, as shown in Figure 2.

Stage 1: By adding nanoscale SCCB (zero-dimensional conductive filler) to the 110-2-type VMQ silicone rubber matrix, a conductive network with basic electrical properties will initially form in the system. In this process, it is necessary to first explore the threshold concentration of SCCB in the system through experiments. Subsequently, further exploration is required to determine the most suitable filling concentration for enhancing material performance after reaching the SCCB threshold concentration.

Stage 2: Based on the adhesive system obtained in the first stage, FMSP (a two-dimensional highly conductive filler) is further added to the system to significantly optimize its electrical properties by utilizing metal conductive fillers. During the preparation process in stage 2, the strong motion characteristics of SCCB in the system can be utilized to help FMSP disperse fully in the system, and the flexible characteristics of the material system can also be optimized.

Stage 3: Based on the rubber compound system formed in the second stage, continue to fill the system with single-walled carbon nanotubes (one-dimensional highly conductive filler). The microstructure of the one-dimensional SWCNTs is shown in the lower-right corner of Figure 2. The characteristic of having a relatively high aspect ratio in size endows them with the ability to change its distribution and orientation with stress variations. Therefore, when the material is in a tensile state, SWCNTs will move to the intermediate region of FMSP along with the molecular chain of the material, forming a new electrical connection phase within the adhesive system. At the same time, it can reduce the attenuation amplitude of the material’s conductivity, thereby achieving the goal of enhancing the conductivity stability of the system.

In the three stages of study, suitable and effective methods need to be utilized to accurately characterize various performance parameters of the material system (including electrical properties, mechanical properties, and microstructural characteristics) to achieve the ultimate goal of optimizing the performance of silicone rubber–based multi-dimensional filler composite electrode materials in stages. The overall work content involves constructing a novel material system based on material formulas A1~A3 while exploring the impact of various multi-dimensional and multi-sized conductive fillers on the performance of electrode materials at different stages.

In this work, the electrode buildup sequence was determined based on interfacial structural optimization and electrochemical performance requirements. Alternative fabrication sequences easily cause interfacial gaps, poor contact, and high impedance, while the adopted sequence can ensure tight layer combination, continuous conductive pathways, and uniform stress distribution, which are beneficial to improving electrode stability and reaction kinetics.

### 2.2. Materials and Formulas

The 110-2-type VMQ is used as the silicone rubber matrix. The following substances were purchased from Macklin (Shanghai, China): n-Heptane standard solution (AR Grade), isopropanol (AR Grade), triallyl isocyanurate (TAIC, ≥98%), and superconductive carbon black (SCCB, 35~40 nm). Single-walled carbon nanotubes (SWCNTs, >95%) and flaky micron-sized silver powder (FMSP, 1~5 μm) were purchased from EFL. Vinyl triethoxysilane (A151, CP Grade) was used as the silane coupling agent. Dicumyl peroxide (DCP, ≥99%) was used as the rubber vulcanizing agent.

The formulas A1~A3 for the three stages of silicone rubber–based multi-dimensional filler composite electrode materials are shown in Table 1 (the amount of material used is expressed in parts per hundred rubber, phr).

In the first stage, the zero-dimensional SCCB nanoparticles were incorporated into the 110-2-type VMQ silicone rubber matrix. The primary purpose of adding SCCB is to construct a preliminary and uniform conductive network inside the insulating rubber matrix. We systematically explored the percolation threshold concentration of SCCB and its optimal filling content. As the foundational conductive filler of the system, SCCB solves the core problem of insulating characteristics of pure silicone rubber and endows the composite with basic electrical conductivity, which is the indispensable premise for the subsequent performance optimization of the composite material.

In the second stage, on the basis of the SCCB foundational conductive system, two-dimensional FMSP was introduced. Relying on the good fluidity and movement characteristics of dispersed SCCB nanoparticles, FMSP can be uniformly dispersed in the rubber matrix without agglomeration. The high-conductivity metal FMSP further optimizes and strengthens the internal conductive network of the material, greatly improving the overall electrical conductivity of the composite. Meanwhile, the synergistic filling of SCCB and FMSP effectively optimizes the flexibility and interfacial compatibility of the rubber system, improving the structural uniformity of the composite.

In the third stage, one-dimensional SWCNTs were filled into the composite system. Different from the foundational conductive function of SCCB and the high-conductivity enhancement function of FMSP, SWCNTs with a high aspect ratio mainly undertake the task of improving the conductive stability of the material under stress deformation. As shown in Figure 2, SWCNTs have a large length–diameter ratio and good orientation-adjustment characteristics. When the material is stretched, SWCNTs can follow the movement of the rubber molecular chains and migrate to the gap regions between FMSP fillers, building new bridging conductive paths. This unique structural advantage effectively compensates for the attenuation of the conductive network caused by structural stretching, reduces the decline range of conductivity, and significantly improves the electromechanical stability of the composite under tensile loading.

The components of the electrode mixture were selected according to their respective functional characteristics. The synergistic effect of active material, conductive agent, and binder is essential for constructing high-performance electrodes. On the basis of preliminary comparative experiments, the optimized component ratio was confirmed to effectively balance electrical conductivity, structural integrity, and electrochemical capacity, exhibiting superior comprehensive performance over other formulas.

### 2.3. Mechanical Mixing Process of Silicone Rubber

The mechanical mixing process of silicone rubber involves utilizing the mechanical shear action of a rubber mixing machine to uniformly mix raw materials. Initially, the rubber compound is introduced into a torque rheometer for heating, softening, and blending modification. The mold cavity temperature is set at 50 °C, and the addition level is 70%. During this process, fillers and vulcanizing agents can be added according to the recipe. Once the raw materials in the torque rheometer are uniformly mixed, the discharge can be completed. Subsequently, on the open mill, the roller gap is adjusted to be narrow, and the rubber is continuously mixed until the color of the rubber compound is uniform without color spots or patches, and there is no internal agglomeration. Here, the roller gap can be widened to complete the discharge, which requires a static placement of 8~16 h.

### 2.4. Vulcanization Process of Silicone Rubber

The mixed rubber compound needs to be further improved through the vulcanization process. First, a vulcanization test is conducted, with the vulcanizer set at a temperature of 170 °C for 30 min, to obtain a vulcanization curve. The vulcanization curve indicates the required optimal cure time T_90_ for the material. Subsequently, the mixed rubber compound is subjected to mold vulcanization based on T_90_. For the first stage of vulcanization, a flat vulcanizer is used, set at 170 °C × T_90_. To ensure that the vulcanizing agent can fully function and achieve effective vulcanization of the rubber compound, a secondary vulcanization operation is further conducted in an oven, set at 200 °C for 4 h.

### 2.5. Surface Modification Process of FMSP

FMSP, due to its unique two-dimensional structure, can provide a larger contact area, which is conducive to enhancing conductivity. Therefore, selecting FMSP and modifying its surface—while determining the optimal addition level and optimizing its dispersion state—are crucial for preparing high-performance electrode materials. First, 35 g of dried FMSP was added to a beaker containing 450 mL of anhydrous ethanol, and heated in a water bath at 70 °C while being stirred with ultrasonic oscillation for 1 h to obtain a 10% mass fraction FMSP solution (uniform state is silver gray). Subsequently, a pipette was used to aspirate a 5% mass fraction coupling agent of FMSP that was added to the solution, followed by continued ultrasonic oscillation and stirring for 2 h while maintaining the water bath temperature at 70 °C. Then, the solvent was dried using a vacuum oven, and the bottom powder was repeatedly filtered and washed using a vacuum filter press. Finally, the vacuum oven was set to 90 °C to dry the filtered powder, obtaining modified FMSP.

### 2.6. Electrical Property Testing Methods

Utilizing the HPS2662 four-point probe resistivity tester (HELPASS Electronic Technology Co., Ltd., Changzhou, China) and its accompanying dedicated correction software, the volume resistivity (Ω·cm) of the material sample can be directly measured. Subsequently, the conductivity σ (S/m) of the sample can be further calculated using Equation (1):

(1) $$\sigma ={\rho_{v}}^{-1}=\frac{h}{RS}$$

where

*R*—the volume resistance of the sample, Ω;

*S*—the effective area of the sample, m^2^;

*h*—the thickness of the sample, m.

During the measurement of the bulk resistivity of a sample using a four-point probe resistivity tester, *R*, *S*, and *h* are based on the effective measurement area covered by the tester’s probe, so they can be treated as constants.

The conductivity stability of electrode materials refers to their ability to maintain excellent and stable conductivity under tensile deformation. During normal operation, the electrode materials are firmly bonded to the surface of the DE material and undergo tensile deformation in accordance with their electro-induced deformation state. Therefore, the ability of electrode materials to maintain excellent conductivity under tensile deformation is a key factor in ensuring the reliable operation of DEAs.

The conductivity of the sample under tensile deformation can be further represented by Equation (2):

(2) $$\mathrm{The}\;\mathrm{rate}\;\mathrm{of}\;\mathrm{change}\;\mathrm{in}\;\mathrm{conductivity}\;=\frac{\Delta \sigma }{\sigma_{0}}\times 100\%$$

### 2.7. Mechanical Property Testing Methods

Appropriate mechanical properties are the fundamental requirements for electrode materials in DEA devices, directly influencing the electro-induced deformation and other properties of the DEA devices. Using traditional tensile methods, corresponding tests can be conducted on samples with the aid of a universal material tensile testing machine to obtain the corresponding stress–strain curve. Furthermore, mechanical property parameters, such as tensile strength, Young’s modulus, tensile elongation at break, and quantitative tensile stress, can be further tested.

The DEA device is a later combination product of electrode materials and DE materials. To ensure that the flexible electrode can adhere tightly to the DE material without slipping or even falling off during operation, the electrode material must possess reliable adhesive properties. The adhesive properties between the electrode material and the DE material are tested using a universal material tensile testing machine (adhesive T-peel strength test), which can directly obtain the adhesive force value of the two-phase interface. Based on Formula (3), the peel strength parameter (bond strength) between the electrode material and the DE material can be further obtained:

(3) $$\sigma_{T}=\frac{F}{W}$$

where

$\sigma_{T}$—the peel strength of the sample, N/m;

*F*—the adhesive force of the sample, N;

*W*—the width of the sample, m.

The electrode material must possess high conformability (high flexibility) to ensure that it does not hinder the electro-induced deformation of the DE material while functioning. The hardness of the electrode material sample is tested using a Shore A durometer. The pre-prepared rubber strip samples are stacked to a thickness of approximately 6–8 mm, the measuring head of the durometer is steadily and quickly pressed down, and the integer result is quickly read within 3 s.

### 2.8. Microscopic Morphology Analysis and Characterization Methods

The macroscopic properties of electrode materials are fundamentally determined by their microscopic morphology. Analyzing the surface morphology, composition, and microstructure of newly designed and prepared electrode materials can help fundamentally enhance their performance. Scanning electron microscopy (SEM) allows for a direct observation of the dispersion state of conductive fillers in the gel, facilitating cross-validation with the designed and hypothesized material morphologies and models.

## 3. Results and Discussion

### 3.1. Stage 1: Performance Testing and Characterization of SCCB/VMQ Composite Electrode Materials

**Figure 3:**
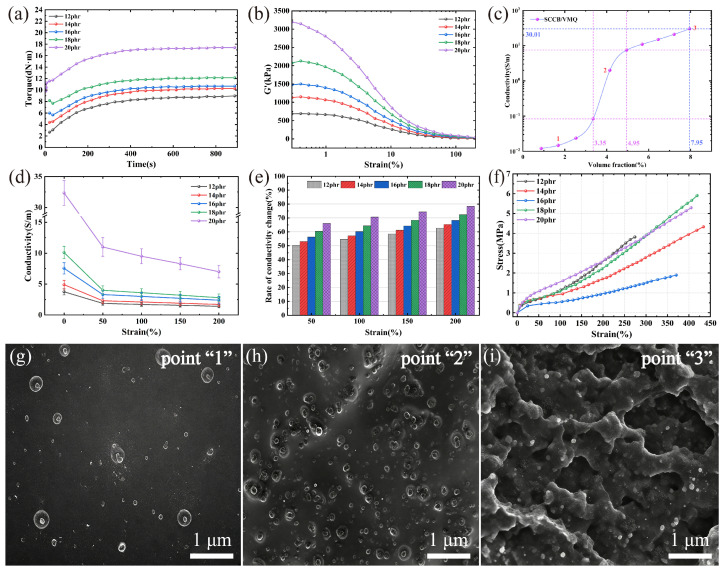
Performance testing and characterization of SCCB/VMQ composite electrode materials: (**a**) the vulcanization curve; (**b**) the RPA test curve; (**c**) the volume fraction–conductivity curve; (**d**) the electrical conductivity under tensile state; (**e**) the stability of conductivity; (**f**) the tensile stress–strain curve; (**g**) the SEM image at point “1”; (**h**) the SEM image at point “2”; (**i**) the SEM image at point “3”.

The processing performance of the SCCB/VMQ composite electrode materials in stage 1 was tested and analyzed using the vulcanometer and rubber processing analyzer (RPA), resulting in the data graphs shown in Figure 3a,b. The following information can be obtained from the vulcanization curve in Figure 3a:

(1) With the increase in the amount of SCCB added to the SCCB/VMQ composite electrode material (12~20 phr, with an interval of 2 phr), the torque value of the material on the vulcanometer also increases (the torque value of the 20 phr material sample is basically twice that of the 12 phr material sample). At the same time, the scorch time (scorch time is a key parameter in the rubber vulcanization characteristic curve, indicating the length of time that the rubber compound maintains fluidity during the vulcanization induction period) also decreases, and the overall processing performance of the material exhibits relatively stable and regular characteristics.

(2) The flat vulcanization zone of the SCCB/VMQ composite electrode material is quite long (the curve tends to flatten after 400 s), indicating its relatively stable processing performance (without over-vulcanization).

The test curve of the rubber processing analyzer in Figure 3b can reflect the dispersion of SCCB conductive fillers in the VMQ matrix (the main reason for the change in storage modulus G′ is the degree of interaction between the fillers and the rubber network within the material system). Furthermore, the following information can be obtained:

(1) With the increase in the amount of SCCB added to the SCCB/VMQ composite electrode material (12~20 phr, with an interval of 2 phr), the storage modulus G′ of the compound gradually increases, indicating a greater degree of interaction between SCCB and the rubber system network;

(2) When the addition level of SCCB is increased from 18 phr to 20 phr, the storage modulus G′ of the material experiences a significant increase (nearly 60%), indicating that when the SCCB addition level reaches 20 phr, it has been able to fill all the empty spaces within the rubber network of the system.

As the rigid SCCB particles are uniformly dispersed in the VMQ matrix, they act as physical cross-linking sites to constrain the movement of rubber molecular chains. A higher filler loading strengthens the interfacial contact and intermolecular interaction between SCCB and the VMQ network, which restricts the deformation of the composite under dynamic shear, and, consequently, leads to a continuous rise in storage modulus. The steady growth of G′ across 12~18 phr demonstrates that SCCB maintains a good dispersion state and forms progressive interfacial bonding with the rubber network.

The sharp rise in storage modulus at 20 phr reveals that the internal voids of the VMQ network are fully occupied by SCCB fillers. Excessive fillers no longer disperse independently but tend to accumulate and form dense filler clusters. This phenomenon further enhances the overall rigidity of the material and greatly limits the flexibility and chain mobility of the rubber matrix. Meanwhile, the remarkable modulus variation also verifies that strong interfacial interaction exists between SCCB and the VMQ network throughout the tested concentration range, which is consistent with the filler dispersion characteristics reflected by the rubber processing analysis curves.

The conductivity of the SCCB/VMQ composite electrode material samples was tested using a resistivity tester, yielding the results shown in Figure 3c. Based on the percolation threshold theory, the percolation range of SCCB is determined to be 3.35% to 4.95% by volume (approximately 8 to 12 phr). Within the percolation threshold, the conductivity of the composite electrode material sample exhibits an exponential growth trend, rapidly increasing from approximately 10^−1^ S/m to nearly 10^1^ S/m. It can be inferred that conductive pathways have initially formed within the electrode material sample, and a conductive network system primarily composed of SCCB has begun to gradually take shape. When the concentration of SCCB filler exceeds the percolation threshold, the conductivity increase of the sample decreases and remains basically in the order of magnitude of 10^1^ S/m. When the volume fraction of SCCB increases from about 4.95% (approximately 12 phr) to about 7.95% (approximately 20 phr), its conductivity increases from approximately 10^1^ S/m to approximately 3 × 10^1^ S/m. It can be concluded that when the concentration of SCCB filler reaches a certain level, the filler accumulates densely in the rubber system, and the conductivity will no longer increase by an order of magnitude.

After crossing the percolation threshold, the conductive network inside the composite has been fully constructed and interconnected. At this stage, the newly added SCCB particles can no longer create a large number of new independent conductive paths, which is the fundamental reason for the slowed growth of conductivity. As the filler loading continues to rise, excessive SCCB particles tend to agglomerate in the VMQ matrix. These dense filler aggregates will introduce tiny gaps and interfacial defects between particle clusters, increasing contact resistance between adjacent conductive units. Although more fillers can slightly optimize the continuity of the existing network and further improve conductivity, the promotional effect is limited. Meanwhile, excessive filler stacking also reduces the deformability of the rubber matrix and deteriorates the dispersion uniformity of SCCB. Therefore, the electrical conductivity only presents a slow linear increase rather than explosive exponential growth, and the overall value stays within the same order of magnitude.

To verify the aforementioned analysis, scanning electron microscopy (SEM) images (20 kV, 20,000×) were taken at points “1”, “2”, and “3” in Figure 3c, as shown in Figure 3g–i. In Figure 3g, the number of SCCB particles is relatively small and sparsely distributed, with no effective connections formed between particles. In Figure 3h, the number of SCCB particles has significantly increased, and a number of conductive pathways have formed in some areas, indicating a significant improvement in conductivity compared to the state shown in Figure 3g. Figure 3i depicts the internal state of the material with a SCCB volume fraction of approximately 7.95% (approximately 20 phr), where conductive particles are densely distributed, forming a relatively complete conductive network. After the SCCB loading reaches the percolation threshold, the overall performance of the composite is further improved with increasing filler content. For the silicone rubber–based flexible electrodes fabricated in this work, the optimal SCCB loading range is 8~12 phr. Within this range, the material achieves high electrical conductivity, excellent flexibility, and low modulus simultaneously. When the filler loading exceeds 12 phr, severe agglomeration of SCCB particles occurs within the matrix. These agglomerates create numerous internal defects, which remarkably increase both the hardness and Young’s modulus of the composite. Consequently, the flexibility and stretchability of the electrode deteriorate, and the conductive network becomes unstable under cyclic deformation. Accordingly, 12 phr is defined as the upper limit for the loading of SCCB for this system. Excessively high filler content will impair the overall electromechanical properties of the flexible electrode.

**Figure 4:**
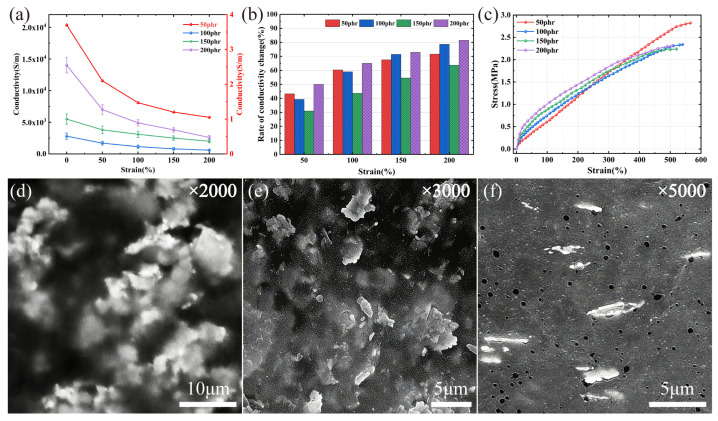
Performance testing and characterization of SCCB/FMSP/VMQ composite electrode materials: (**a**) the electrical conductivity under tensile state; (**b**) the stability of conductivity; (**c**) the tensile stress–strain curve; (**d**) the SEM image at 2000×; (**e**) the SEM image at 3000×; (**f**) the SEM image at 5000×.

Based on the above analysis, subsequent research should be conducted on samples with SCCB addition levels ranging from 12 to 20 phr. Figure 4d,e present the results of further electrical property tests on the material, leading to the following conclusions and targeted optimization strategies for material electrical performance:

(1) The conductivity of the composite electrode material samples in their initial state increases with the increase in the amount of SCCB added.

(2) When the tensile strain of each group of samples reached 50%, their electrical conductivity had significantly decreased, with the change rate of conductivity exceeding 50%. When the tensile strain of the samples exceeded 50% (50% to 200%), their electrical conductivity still exhibited a slight linear decrease, but remained stable overall. Furthermore, the higher the amount of SCCB added, the greater the decrease in the electrical conductivity of the samples.

(3) The conductive network formed by SCCB has been largely destroyed under tensile strain within 50%, resulting in severe degradation of the material’s conductivity. The sharp damage of the conductive network under low strain is the key bottleneck restricting the electromechanical stability of the composite material.

(4) Combined with the above electrical variation rules, the electrical performance of the composite material can be further optimized in a targeted manner. The core optimization approach is to enhance the damage resistance and self-healing ability of the internal SCCB conductive network under low-strain conditions (less than 50%). Specifically, the interfacial compatibility between SCCB filler and the polymer matrix can be improved via filler modification and matrix optimization to build a flexible, stable, and damage-resistant conductive network. This strategy can effectively suppress the rapid collapse of the conductive network under low tensile deformation, reduce the sharp attenuation of conductivity, and, ultimately, improve the electromechanical coupling stability and service reliability of the composite electrode material under tensile working conditions.

To further evaluate the conductive fatigue stability of the prepared electrodes under repeated deformation, a 200-cycle tensile cyclic test was supplemented. The electrical performance of the sample remained stable during cyclic loading without obvious degradation. Accordingly, the cyclic test data are displayed as error bars in Figure 3d, which quantitatively characterize the fatigue resistance and long-term electrical stability of the conductive network under cyclic actuation.

Figure 3f and Table S1 present the data obtained from the mechanical property tests conducted on SCCB/VMQ composite electrode material samples. The following conclusions can be drawn:

(1) When the SCCB filling content ranges from 12 phr to 20 phr, the material samples generally exhibit extremely high tensile elongation at break (>400%) and a relatively low Young’s modulus (<4 MPa), indicating that the materials within this range possess good flexibility characteristics overall.

(2) The hardness of the material samples shows an increasing trend with the increase in SCCB filling content, and the rate of increase gradually becomes larger. Additionally, the tensile fracture permanent-deformation rate of the samples also exhibits an increasing trend. Comprehensive analysis of the above situation indicates that a higher SCCB filling content, while beneficial for the construction of the conductive network, can damage the flexible characteristics of the material.

(3) Through comprehensive comparative analysis of the performance of samples with SCCB filler contents ranging from 12 to 20 phr, it can be concluded that the 12 phr SCCB/VMQ composite electrode material sample better meets the performance requirements of DEAs for flexible electrode materials. Furthermore, the 12 phr sample still retains considerable internal space for further research on multi-dimensional filler applications.

### 3.2. Stage 2: Performance Testing and Characterization of SCCB/FMSP/VMQ Composite Electrode Materials

Based on the test results in Section 3.1, the electrode material with an SCCB addition level of 12 phr was selected for further research on multi-dimensional filler composite electrode materials. Electrical performance tests were conducted on samples of the SCCB/FMSP/VMQ composite electrode materials, resulting in the data graphs shown in Figure 4a,b. The following conclusions can be drawn:

(1) When the addition of FMSP is within 50 phr, there is no trend of improvement in the conductivity of the new material sample. This indicates that 50 phr of FMSP does not generate a new conductive network within the rubber compound.

(2) When the addition of FMSP exceeds 50 phr, the conductivity of the new electrode material exhibits a significant improvement trend, surging from the order of magnitude of 10^0^ S/m to 10^3^ S/m and further reaching the order of magnitude of 10^4^ S/m. This indicates that FMSP above 50 phr generates a new and more effective conductive network within the compound.

(3) Compared to samples without FMSP, the conductivity change rate of the new material samples at 50% tensile strain showed a certain degree of decrease (none exceeding 50%). This indicates that FMSP has optimized the composition of the conductive network within the new adhesive system, effectively enhancing the conductive stability of the composite electrode material.

(4) When the addition level of FMSP is at 150 phr, the sample exhibits the best conductivity stability, with a conductivity degradation of only about 30.9% under 50% tensile strain. This indicates that FMSP possesses a distinctive percolation threshold, which endows its internal conductive network with higher stability and reliability.

A 200-cycle repeated tensile test was further carried out to verify the cyclic fatigue stability of the conductive network. The results show that the composite electrode possesses stable electrical performance after long-term cyclic deformation. The corresponding cyclic data are presented as error bars in Figure 4c, which intuitively reflect the dispersion and stability of conductivity under cyclic deformation conditions.

Figure 4c and Table S2 present the mechanical property tests conducted on the SCCB/FMSP/VMQ composite electrode material samples. To further clarify the regulatory effect of FMSP on the mechanical flexibility of composite materials and its offsetting mechanism against the adverse mechanical effect of SCCB, the in-depth analysis based on test data is summarized as follows:

(1) The A2 formula sample, which incorporates FMSP based on the A1 formula, generally exhibits an extremely high tensile elongation at break (>500%) and a low Young’s modulus (<4 MPa). This indicates that the electrode material based on the new formula generally possesses good flexibility characteristics. The low-modulus and high-ductility features are essential for flexible electrode materials to adapt to repeated bending, stretching, and other deformation scenarios.

(2) Compared to the A1 formula sample, the A2 formula sample exhibits improved tensile elongation at break (increased from 400%+ to 500%+) and bonding performance. Furthermore, the tensile strength, quantitative tensile stress at 100% and 300%, and hardness of the new sample have significantly decreased. Rigid SCCB fillers tend to restrict the slippage and rearrangement of VMQ molecular chains, which increases material stiffness and reduces ductility, inducing adverse effects on the flexible mechanical properties of the composite. The addition of FMSP can effectively offset this negative effect: FMSP optimizes the internal filler network structure and interfacial interaction, relieves the rigid constraint caused by SCCB, endows the composite system with better molecular chain mobility, and significantly improves the flexible deformation ability of the material. Nevertheless, the introduction of FMSP also weakens the cross-linking degree of the internal network of the compound, resulting in an increased permanent set at break.

(3) The composite electrode material sample with an FMSP addition of 150 phr exhibits the lowest tensile set percentage (13%), indicating that the dispersion and mixing state of the various conductive fillers and collective components within this sample is optimal compared with the other experimental groups, enabling the sample to achieve maximum self-recovery after extensive tensile deformation. It further demonstrates that a reasonable FMSP dosage can effectively coordinate the filling state of SCCB, eliminate the mechanical defect points caused by uneven distribution of rigid fillers, balance the mechanical contradiction between stiffness and flexibility, and realize the optimal flexible mechanical stability of composite electrode materials.

Figure 4d–f present SEM images (20 kV, at 2000×, 3000×, and 5000× magnification) of the cross-section of the SCCB/FMSP/VMQ composite electrode material samples based on the A2 formula—with an SCCB addition of 12 phr and an FMSP addition of 150 phr—at different magnifications. As shown in Figure 4d, at a magnification of 2000×, the FMSP filler has dispersed and spread within the system, forming a number of conductive pathways without significant structural defects. This indicates that the two-dimensional FMSP filler performs well, and, with the assistance of the A151 silane coupling agent, collectively forms a stable and good compatibility state with VMQ. Figure 4e reveals that at a magnification of 3000×, the SCCB filler particles and aggregates are relatively evenly dispersed among the FMSP particles. Figure 4f shows that at a magnification of 5000×, FMSP is dispersed in a flat-like manner, with uniformly distributed SCCB particles in the spacing areas. The electrode material states exhibited in Figure 4d–f are consistent with the second-stage functional design model in Section 2.1, indicating that the two-dimensional FMSP filler plays a role in enhancing the electrical properties of the electrode material at this stage. Based on the experimental test results in this section, the following conclusions can be drawn:

(1) The new composite electrode material with FMSP filler added to the SCCB/VMQ composite electrode material system exhibits significant improvements in both conductivity and conductivity stability, proving that the two-dimensional FMSP conductive filler indeed possesses excellent conductivity.

(2) The addition of FMSP filler has also improved the mechanical properties of the material to a certain extent, such as the decrease in elastic modulus, indicating that its flexibility has been enhanced.

(3) Through comparative analysis, it can be concluded that the composite electrode material sample with an FMSP addition of 150 phr (and an SCCB addition of 12 phr) exhibits the best electrical conductivity stability and overall optimal mechanical properties. Therefore, it is selected as the research subject for further multi-dimensional filler experiments in the next stage.

### 3.3. Stage 3: Performance Testing and Characterization of SCCB/FMSP/SWCNT/VMQ Composite Electrode Materials

**Figure 5:**
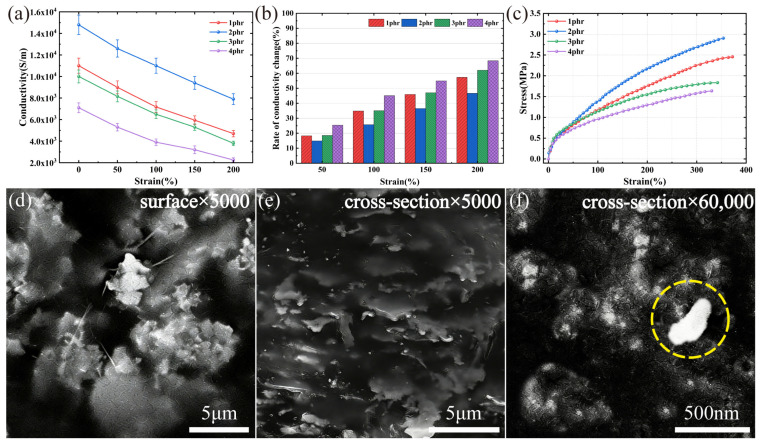
Performance testing and characterization of SCCB/FMSP/SWCNT/VMQ composite electrode materials: (**a**) the electrical conductivity under tensile state; (**b**) the stability of conductivity; (**c**) the tensile stress–strain curve; (**d**) the SEM image of the sample surface at 5000×; (**e**) the SEM image of the sample cross-section at 5000×; (**f**) the SEM image of SWCNT particles at 60,000×.

Based on the test results in Section 3.2, the electrode material with an SCCB addition of 12 phr and an FMSP addition of 150 phr was selected for further research on multi-dimensional filler composite electrode materials. The electrical performance tests conducted on the SCCB/FMSP/SWCNT/VMQ composite electrode material samples yielded the data graphs shown in Figure 5a,b. The relevant mechanism of conductivity variation and the conductive network evolution are further analyzed, and the detailed conclusions are summarized as follows:

(1) The conductivity of the material significantly improves after the introduction of SWCNTs, which essentially benefits from the formation of a new and more efficient ternary conductive network inside the adhesive. The original binary conductive network composed of SCCB and FMSP has numerous microscopic gaps and discontinuous conductive channels. The one-dimensional SWCNTs with high aspect ratio can uniformly disperse in the matrix, fill the structural voids of the binary network, and act as a conductive bridge between discrete fillers. This synergistic effect eliminates conductive dead zones, reduces internal contact resistance, and constructs a more continuous and efficient conductive pathway system, thereby realizing the improvement in macroscopic conductivity.

(2) When the addition of SWCNTs is within 2 phr, the conductivity of the new sample increases with the increase in SWCNTs; when the addition exceeds 2 phr, the conductivity tends to decrease. This indicates that there is a “percolation threshold” for SWCNTs to enhance the conductivity of the material. According to the test results, 2 phr is the optimal addition level of SWCNTs. Below the percolation threshold, the continuous access of SWCNTs optimizes the ternary conductive network structure and improves conductive efficiency; excessive SWCNTs will induce serious nanofiller agglomeration, destroy the uniformity of the conductive network, and introduce structural defects, resulting in reduced electrical conductivity.

(3) The addition of SWCNTs significantly improved the conductive stability of the new material, with the conductive performance degradation under 50% tensile strain being less than 26%, with the best value being only 14.9%. This indicates that, after SWCNTs are uniformly dispersed in the adhesive, they can fill the conductive network gaps left by FMSP and SCCB, especially when the sample is in a tensile state, where the original conductive network gaps increase or even break. The bridged SWCNT conductive channels can effectively compensate for the failure of partial binary conductive pathways, maintain the integrity of the overall conductive network under tensile deformation, and greatly enhance the electromechanical stability of the material.

(4) Although the conductivity stability of the new material has been improved, the improvement effect is limited. This indicates that excessive addition of SWCNTs hinders the rubber molecular fluidity of the material sample under large tensile strain, making it difficult to recover the internally damaged conductive network. Excess nanofillers will restrict the movement and rearrangement of VMQ molecular chains, reduce the flexibility and self-recovery ability of the conductive network, and ultimately limit the further improvement in the material’s electromechanical performance.

To verify the long-term service reliability of the optimized composite electrode, 200 cyclic tensile tests were performed. No significant conductivity attenuation was observed after repeated deformation. The cyclic stability data are supplemented as error bars in Figure 5a, which effectively demonstrate the excellent cyclic fatigue resistance of the optimized SCCB/FMSP/SWCNT synergistic conductive network.

Figure 5c and Table S3 present the data obtained from the mechanical property tests conducted on the SCCB/FMSP/SWCNT/VMQ composite electrode material samples. Based on the analysis, the following conclusions can be drawn:

(1) The Shore hardness of the A3 formula sample remains essentially unchanged compared to the A2 sample, but there is a noticeable increase in Young’s modulus, and the elongation at break of all types of samples has a significant decrease (500%+~300%+). This indicates that the addition of SWCNTs has a certain degree of reinforcement effect on the overall mechanical properties of the compound, but it also reduces the flexibility characteristics of the material.

(2) The permanent set rate of the A3 formula sample increases with the increase in the amount of SWCNTs added, indicating that when the sample is in a tensile state, the SWCNT particles will follow the orientation of the internal rubber network of the material; after the external force is removed, the SWCNT particles, whose orientation has changed, hinder the recovery movement of the rubber network. In summary, due to the high aspect ratio of SWCNTs, which makes them prone to movement with tensile orientation, excessive addition is not suitable. According to testing, 2 phr is the optimal amount to add.

(3) Although the elongation at break of sample A3 decreases sharply from 520% (A2) to 354%, this reduction will not affect the practical performance of the DEA. In the actual working process of the soft robot, the maximum uniaxial actuation strain of the prepared DEA is less than 50%, which is far below the measured elongation at break of A3. Consequently, the stretchability of A3 is fully adequate to satisfy the operational requirements of the device, and, thus, this index is not further discussed in this work.

Figure 5d–f present the SEM images of the SCCB/FMSP/SWCNT/VMQ composite electrode material samples based on the A3 formulation, with an SCCB addition of 12 phr, an FMSP addition of 150 phr, and an SWCNT addition of 2 phr. To intuitively and comprehensively explain the performance of the samples from a microscopic perspective, the surface and cross-section of the samples were observed using SEM. The observation conditions were set as follows: 5000× magnification for the sample surface, 5000× magnification for the sample cross-section, and 60,000× magnification for SWCNTs.

Figure 5d depicts the surface morphology of the electrode material sample after a 50% stretching treatment under 5000× magnification. It can be observed that the SWCNTs are now interlaced within the gaps of the FMSP, forming a conductive network pathway, which aids in enhancing the conductivity stability of the material sample under tensile strain conditions within 50%. However, a portion of the newly formed “carbon–silver” connection structure between SWCNTs and FMSP replaces the original “silver–silver” metal connection structure between the FMSP, resulting in a certain degree of degradation in conductivity, consistent with the performance test results and analytical inferences from the previous two sections. Figure 5e illustrates the cross-sectional morphology of the stretched and fractured electrode material sample under 5000× magnification. It can be observed that the distribution and orientation of the FMSP and SWCNTs within the sample after stretching adjust according to the stress direction, so the two-dimensional FMSP filler only exhibits one-dimensional geometric morphological characteristics as “lines” in Figure 5e. Meanwhile, the originally one-dimensional SWCNTs specifically manifest as zero-dimensional geometric morphological states as “bright spots”, corresponding to the situation in Figure 5f. Figure 5f depicts the morphological state of SWCNTs in the cross-section under 60,000× magnification, accompanied by clusters of SCCB particles. Figure 5e,f demonstrate that, in this state, SWCNTs and SCCB are dispersed around and within the interlayers of FMSP, consistent with the third-stage functional design model described in Section 2.1.

It should be noted that the microscopic characterizations in this work are all based on post-fracture static observations, which only reflect the final distribution state of SWCNT, FMSP, and SCCB fillers after tensile deformation. The real-time dynamic migration, orientation evolution, and conductive network reconstruction behavior of fillers at different tensile strains cannot be directly captured. Accordingly, the proposed stage 3 mechanism, namely the migration of SWCNTs toward the intermediate region of the FMSP to optimize the conductive network, is concluded and inferred by combining macroscopic electrical and mechanical test results with static microscopic morphology analysis. In situ tensile SEM tests and systematic strain-dependent microscopic characterization at multiple elongation states have not been conducted in the present study. In follow-up work, we will cooperate with external scientific research platforms to carry out in situ dynamic microscopic tests to continuously monitor the dynamic evolution of filler distribution and the conductive network structure during stretching. Further quantitative analysis will be performed to verify the migration mechanism of SWCNTs and refine the microscopic interpretation of the performance variation in composite electrodes.

### 3.4. Performance Analysis and Comparison of Silicone Rubber–Based Multi-Dimensional Filler Composite Electrode Materials

**Figure 6:**
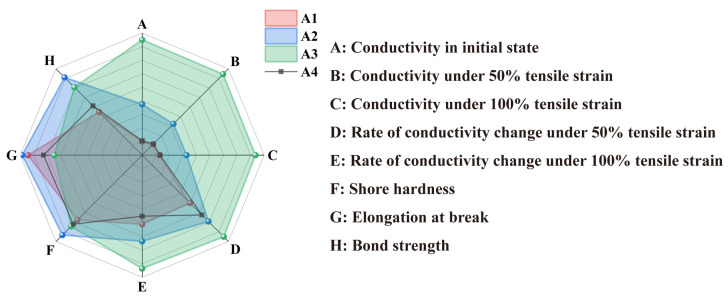
Comparison of main properties of optimal samples in three stages of silicone rubber–based multi-dimensional filler composite electrode materials.

Figure 6 and Table S4 comprehensively analyze and compare the main performance indicators of the composite electrode material samples with optimal performance in three stages. A sample labeled A4, filled with only 2 phr SWCNTs, was set as the control group. (A1: SCCB 12 phr; A2: SCCB 12 phr/FMSP 150 phr; A3: SCCB 12 phr/FMSP 150 phr/SWCNTs 2 phr; A4: SWCNTs 2 phr). The staged variation characteristics of electrical conductivity and conductive stability are closely correlated with the graded functional design model of the composite materials, which further verifies the effectiveness of the multi-dimensional filler complementary synergistic design concept. The detailed conclusions are summarized as follows:

(1) The conductivity of the silicone rubber–based multi-dimensional filler composite electrode material samples has been optimized and improved in stages, from the order of magnitude of 10^0^ S/m in A1 to the order of magnitude of 10^3^ S/m in A2, and further to the order of magnitude of 10^4^ S/m in the third stage. The progressive enhancement of conductivity fully matches the core connotation of the graded functional design model. The staged design of “single-filler basic conductivity construction (A1)—binary-filler network optimization (A2)—ternary multi-dimensional filler high-efficiency conductivity reconstruction (A3)” is realized in terms of macroscopic electrical properties. The step-by-step increase in conductivity corresponds to the gradual improvement and perfection of the internal conductive network structure, which strongly indicates that the complementary interaction of zero-dimensional SCCB, two-dimensional FMSP, and one-dimensional SWCNTs produces a positive synergistic conductive effect. This staged performance evolution fundamentally validates the innovative design idea of improving the comprehensive conductive performance of materials through multi-dimensional filler complementary optimization.

(2) The conductivity stability of the silicone rubber–based multi-dimensional filler composite electrode material samples has been optimized and improved stage by stage. The conductivity of the A3 sample decreased by only 14.9% under 50% tensile strain, and decreased by only 25.7% under 100% tensile strain. The conductivity attenuation of the A3 sample in the third stage under tensile conditions was much lower than that of A1 and A2, indicating that the characteristic of one-dimensional SWCNTs with a high aspect ratio enables them to distribute and orientate with stress changes—that is, to move to the intermediate region of the FMSP along with the molecular chain movement of the material, forming new electrical connection phases within the rubber system. This dynamic complementary conductive mechanism compensates for the structural defects of the traditional binary conductive network under tensile deformation, effectively reduces the attenuation of conductivity under tensile strain conditions, and realizes the progressive improvement in the system’s conductive stability, which further supplements and improves the functional advantages of the multi-dimensional graded design model.

(3) There are no significant differences in hardness, tensile strength, and modulus at a given extension among the silicone rubber–based multi-dimensional filler composite electrode materials: A2 exhibits the best tensile elongation at break (520%) and bonding strength (63 N/cm), but its electrical properties are significantly inferior to those of A3. This indicates that the graded introduction of multi-dimensional fillers can balance the mechanical flexibility and electrical conductivity of the material. FMSP primarily optimizes the mechanical flexibility and interfacial bonding performance of the material, while the ternary compounding of SWCNTs further breaks through the electrical performance bottleneck on the premise of maintaining excellent mechanical properties, realizing the targeted functional optimization of each stage of the graded design.

(4) The radar chart provides an intuitive and clear comparison of the main performance indicators of the four samples. It is observed that the shape of the performance indicators for A3 is closest to the background pattern of a regular octagon and covers the largest area, indicating that the comprehensive performance of A3 is the best among all the samples of silicone rubber–based multi-dimensional filler composite electrode materials at various stages. The optimal comprehensive performance of A3 fully demonstrates that the multi-dimensional filler complementary design model can organically integrate the advantages of different dimensional fillers, overcome the performance limitations of single- and binary-filler systems, and, finally, achieve the simultaneous optimization of electrical conductivity, electromechanical stability, and mechanical flexibility. This fully verifies the feasibility and advancement of the graded functional design and multi-dimensional complementary conductivity enhancement strategy.

(5) To verify the necessity of the synergistic effect of multi-scale fillers, a composite electrode (A4) filled with only 2 phr SWCNTs was additionally prepared as the control group. Overall, the initial electrical conductivity of A4 was markedly higher than that of A1, but much lower than that of the ternary composite system consisting of SCCB, FMSP, and SWCNTs (A3). Meanwhile, A4 exhibited inferior conductive stability compared with A3. Under a tensile strain of 200%, its conductivity attenuation rate reached 82.5%, while the value of A3 was only 46.6%. In addition, A4 showed no obvious advantages in mechanical properties and adhesion performance, with overall performance far below that of A3. These results clearly demonstrate that although SWCNTs can improve the basic conductivity, they cannot replace SCCB and FMSP in constructing stable conductive networks, optimizing interfacial compatibility, and enhancing electromechanical stability. The gradient synergistic effect of the three types of fillers is the key to achieving excellent comprehensive performance of the composite.

(6) Sample A4 has a conductivity intermediate between A1 and A2. It achieves better electrical performance than A1 (SCCB) but worse than A2 (FMSP). When stretched, the conductive network of the SWCNT-only system (A4) easily breaks down because of inhomogeneous filler distribution and the lack of bridging by SCCB and FMSP. A4 shows a larger conductivity loss than A3 but a smaller one than A1, verifying that SCCB and FMSP are critical for improving conductive stability. SWCNTs offer moderate reinforcement to silicone rubber, making the hardness and Young’s modulus of A4 slightly higher than A1 and lower than A3. Due to the absence of synergistic toughening from FMSP and SCCB, A4 has a reduced elongation at break relative to A1 and A2. The SWCNT-filled system also has inferior interfacial compatibility to the SCCB/FMSP composite. Consequently, the adhesion strength of A4 lies between A1 and A3, and is far below A2, which has the best interfacial performance. As a key filler, SWCNTs build conductive networks and enhance electrical conductivity. Their high aspect ratio and adjustable stress orientation also help stabilize conductivity during deformation. Even so, satisfactory overall performance cannot be achieved using SWCNTs alone. SCCB and FMSP are indispensable and undertake distinct tasks in forming basic conductive networks, raising overall conductivity, and sustaining conductive stability under deformation. The excellent performance of the composite is attributed to the synergism of multi-scale fillers, instead of any single constituent.

### 3.5. Fabrication and Experiment of the Micro-Crawling Robot Driven by a DEA Based on Silicone Rubber–Based Multi-Dimensional Filler Composite Electrode Materials

**Figure 7:**
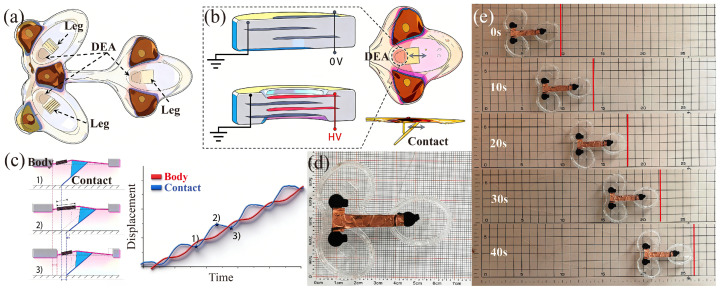
The micro-crawling robot driven by DEA based on silicone rubber–based multi-dimensional filler composite electrode materials: (**a**) the overall structure of the micro-crawling robot; (**b**) the functional structure of DEAs; (**c**) the coordinated motion mode of the micro-crawling robot; (**d**) the prototype of the micro-crawling robot; (**e**) the linear motion performance test. (Figure a–c are reproduced from Ref. [44], Copyright 2019, with permission from AAAS.)

The overall structure of the dielectric elastomer–driven micro-crawling robot based on the silicone rubber–based multi-dimensional filler composite electrode material is shown in Figure 7a, referring to the structures reported in the literature [44]. The body part is mainly composed of a flexible frame, a DEA, and three legs, which utilize directional friction for movement. The front legs are on the symmetrical central axis, while the rear left and right legs are symmetrically distributed off the central axis. The angle between the left and right legs is 90°; each forms a 45° angle with the central symmetrical axis. The robot’s body frame is flexible and can easily deform to adjust its configuration.

The three legs of the micro-crawling robot are driven by the DEA, as shown in Figure 7b. The foot end of each leg contacts the ground at an angle of approximately 45°, suitable for generating directional friction on most surfaces. The DEA consists of alternating layers of three DE films and four layers of silicone rubber–based multi-dimensional filler composite electrode material. Under the drive of an external voltage signal, the dielectric elastomer actuator is activated and drives the corresponding leg to move. The coordinated work of the three legs enables the robot to produce motion behaviors.

The motion principle of the micro-crawling robot is illustrated in Figure 7c. Forward movement is achieved by driving all three legs with the same signal. A single DEA drive cycle and the corresponding leg movement process consist of two steps. In the first step, from state 1 to state 2, an electrical signal is applied, causing the DEA to expand in area and propel the leg forward. In the second step, from state 2 to state 3, the electrical signal is turned off, the DEA reverts to its original state, and the leg is pulled back. Due to the 45° contact angle and directional friction, the legs of the micro-crawling robot remain almost stationary at this point, and the contraction of the DEA relatively pulls the robot’s body forward. Under the influence of a stable electrical signal, the legs of the micro-crawling robot move in a fixed cycle, and the relationship between the displacement of its body and foot ends and time is roughly as shown in the right part of Figure 7c.

Based on the functional structural design scheme of the micro-crawling robot, a prototype of the micro-crawling robot, as shown in Figure 7d, was constructed using fully flexible materials. The body frame was cut from acrylic sheets, and the legs were cut from PET film. The overall weight of the prototype is approximately 2 g, with a maximum size not exceeding 7 cm. It features a flexible frame and a compliant driving structure, and can maintain its normal structure even under certain external force disturbances. Multiple sets of high-voltage electrical signals with different amplitudes and frequencies were applied to the micro-crawling robot prototype to test its optimal motion performance. When a sawtooth-wave electrical signal with an amplitude of 2 kV and a frequency of 10 Hz was applied by Aigtek ATA-7100 (Aigtek Electronic Technology Co., Ltd., Xi’an, China), its motion performance was optimal, with a linear motion speed of approximately 8 mm/s. The motion process is shown in Figure 7e.

## 4. Conclusions

In this work, we systematically investigated the working mechanism of conductive fillers with varied dimensions and particle sizes within silicone rubber matrices across three stages. This study mainly focuses on the electrical conductivity and conductivity stability of silicone rubber composites reinforced with multi-dimensional fillers. The underlying causes for performance evolution are analyzed, and the corresponding variation trends are summarized. We also characterized the dispersion of differently sized conductive fillers in the rubber matrix at each stage, explored their effects and correlations with the mechanical properties of the composite electrodes, and determined the optimal loading content for each filler. Finally, we fabricated a novel silicone rubber composite electrode with multi-dimensional fillers that integrates superior electrical and mechanical performance.

(1) Experimental results reveal that the percolation threshold of SCCB in the A1 formula SCCB/VMQ composite electrodes ranges from 3.35 vol% to 4.95 vol% (equivalent to 8~12 phr by mass). The saturated conductivity of the composite reaches 30.01 S/m at the maximum loading of SCCB.

(2) Three conductive fillers, namely SCCB, SWCNTs, and FMSP, were incorporated into the VMQ matrix to construct a multi-dimensional conductive network. The as-prepared composite electrode exhibits outstanding overall performance. For the optimized sample A3, its electrical conductivity is about 1.5 × 10^4^ S/m, which only drops by 14.9% to around 1.3 × 10^4^ S/m under 50% tensile strain.

(3) Using the A3 electrode material, we assembled a DEA-driven micro-crawling robot prototype, which achieves a maximum linear moving speed of 8 mm/s.

In DEA devices, flexible electrodes function like the vascular system to transmit electrical energy throughout the entire device. While the rapid advancement of flexible electronics in recent years has greatly promoted the innovation of novel flexible conductive materials, several key issues still require further in-depth investigation:

(1) Superior Stretchability and Resilience: DEA devices are designed to undergo regular shape transformation to meet practical structural and dynamic demands, and experience frequent cyclic deformation during service. This imposes strict requirements on the stretchability and shape recovery capability of flexible electrode materials.

(2) Conductive Network Stability: The stability of internal conductive networks is critical to the reliable operation of DEA devices. It covers both fabrication reproducibility and intrinsic material performance, ensuring that the electrodes remain electrically stable under tension, bending, compression, folding, and other mechanical loads.

(3) Interfacing with Rigid Electronic Components: To realize full system functionality, the conductive networks of flexible DEA systems need to be connected with external electronic components. Since most commercial electronic components are rigid, optimizing the interconnection techniques between flexible conductive networks and rigid elements is essential for enhancing the overall system stability under tensile deformation.

## Figures and Tables

**Table 1:** The formulas for the three stages of silicone rubber–based multi-dimensional filler composite electrode materials.

			Materials	Mass Fraction/phr
A3	A2	A1	VMQ	100
			SCCB	10~20
			DCP	2
			TAIC	2
			FMSP	50~200
			A151	6
			SWCNTs	1~4
			VMQ	100

## Data Availability

The original contributions presented in this study are included in the article/Supplementary Materials. Further inquiries can be directed to the corresponding authors.

## Supplementary Materials

The following supporting information can be downloaded at: https://www.mdpi.com/article/10.3390/polym18131561/s1, Table S1: The mechanical properties of SCCB/VMQ composite electrode materials; Table S2: The mechanical properties of SCCB/FMSP/VMQ composite electrode materials; Table S3: The mechanical properties of SCCB/FMSP/SWCNT/VMQ composite electrode materials; Table S4: Comparison of main properties of optimal samples in three stages of silicone rubber–based multi-dimensional filler composite electrode materials.
