# Supplementary material for: Novel Silicone Rubber–Based Multi-Dimensional Filler Composite Electrode Materials for the Dielectric Elastomer Actuation Technology of Micro-Crawling Robots
Source: Polymers (Basel). 2026 Jun 23;18(13):1561. doi: 10.3390/polym18131561 (PMC13363996; doi:10.3390/polym18131561)
Supplement: Supplementary file 1 [file polymers-18-01561-s001.zip › polymers-4337155-supplementary.pdf]

Supplementary Materials for

Novel Silicone Rubber-Based Multi-Dimensional Filler Composite Electrode Materials for the Dielectric Elastomer Actuation Technology of Micro-Crawling Robots

Yang Hong[1,2], Yun Yang[1,2], Zening Lin[1,2], Tao Jiang[1,2*], Zirong Luo[1,2*]

[1] College of Intelligence Science and Technology, National University of Defense Technology, Changsha, Hunan Province, 410073, China

[2] National Key Laboratory of Equipment State Sensing and Smart Support, National University of Defense Technology, Changsha, Hunan Province, 410073, China

* Corresponding author: jiangtao@nudt.edu.cn (T.J.); luozirong@nudt.edu.cn (ZR.L.)

1. The mechanical properties of SCCB/VMQ composite electrode materials

Table S1. The mechanical properties of SCCB/VMQ composite electrode materials

Mechanical property parameters	Addition amount of SCCB/phr				
	12	14	16	18	20
Shore A hardness	30	33	37	47	60
Young's modulus/MPa	1.7	1.9	2.2	2.8	3.5
Tensile strength/MPa	3.7	4.4	5.0	6.0	5.4
Elongation at break/%	490	475	467	460	445
Permanent deformation rate at tensile fracture/%	10	14	17	20	22
Stress for 100% quantitative tensile state/MPa	1.0	0.9	0.9	1.0	0.9
Stress for 300% quantitative tensile state/MPa	3.0	2.7	2.9	3.5	3.5
Bond strength (N/cm)	35	34	32	29	28

2. The mechanical properties of SCCB/FMSP/VMQ composite electrode materials

Table S2. The mechanical properties of SCCB/FMSP/VMQ composite electrode materials

Mechanical property parameters	Addition amount of FMSP/phr			
	50	100	150	200
Shore A hardness	30	31	37	43
Young's modulus/MPa	1.3	1.8	2.9	3.2
Tensile strength/MPa	2.8	2.3	2.2	2.3
Elongation at break/%	565	540	520	505
Permanent deformation rate at tensile fracture/%	29	20	13	26
Stress for 100% quantitative tensile state/MPa	0.6	0.7	0.9	1.0
Stress for 300% quantitative tensile state/MPa	1.7	1.6	1.7	1.8
Bond strength (N/cm)	50	56	63	57

3. The mechanical properties of SCCB/FMSP/SWCNT/VMQ composite electrode materials

Table S3. The mechanical properties of SCCB/FMSP/SWCNT/VMQ composite electrode materials

Mechanical property parameters	Addition amount of SWCNT/phr			
	1	2	3	4
Shore A hardness	31	33	34	36
Young's modulus/MPa	3.0	3.9	4.7	5.4
Tensile strength/MPa	2.5	2.9	1.8	1.6
Elongation at break/%	372	354	342	330
Permanent deformation rate at tensile fracture/%	15	16	18	20
Stress for 100% quantitative tensile state/MPa	1.2	1.4	1.2	1.0
Stress for 300% quantitative tensile state/MPa	2.2	2.7	1.8	1.6
Bond strength (N/cm)	58	55	49	45

4. Comparison of main properties of optimal samples in three stages of silicone rubber-based multi-dimensional fillers composite electrode materials

Table S4. Comparison of main properties of optimal samples in three stages of silicone rubber-based multi-dimensional fillers composite electrode materials

Property parameters			A1	A2	A3	A4
Electrical properties	Electrical conductivity-Conductivity (S/m)	Initial state	3.8	5.5×10^3	1.5×10^4	1.2×10^2
		Tensile strain 50%	1.9	3.8×10^3	1.3×10^4	75.1
		Tensile strain 100%	1.7	3.1×10^3	1.1×10^4	48.2
		Tensile strain 150%	1.6	2.5×10^3	9.4×10^3	32.5
		Tensile strain 200%	1.4	2.0×10^3	7.9×10^3	21.3
	Stability of conductivity-Rate of conductivity change/%	Tensile strain 50%	50.1	30.9	14.9	37.7
		Tensile strain 100%	54.7	43.6	25.7	60.0
		Tensile strain 150%	58.4	54.6	36.5	73.3
		Tensile strain 200%	62.7	63.6	46.6	82.5
Mechanical properties	Hardness	Shore A hardness	30	37	33	32
	Tensile mechanical properties	Young's modulus/MPa	1.7	2.9	3.9	2.1
		Tensile strength/MPa	3.7	2.2	2.9	3.3
		Elongation at break/%	490	520	354	410
		Permanent deformation rate at tensile fracture/%	10	13	16	12
		Stress for 100% quantitative tensile state/MPa	1.0	0.9	1.4	1.1
		Stress for 300% quantitative tensile state/MPa	3.0	1.7	2.7	2.2
	Adhesive performance	Bond strength (N/cm)	35	63	55	40
